# Regulation of TRPM7 Function by IL-6 through the JAK2-STAT3 Signaling Pathway

**DOI:** 10.1371/journal.pone.0152120

**Published:** 2016-03-24

**Authors:** Aifen Liu, Fengbo Zhao, Jing Wang, Yin Zhao, Zhenzhao Luo, Yan Gao, Jing Shi

**Affiliations:** 1 Department of Neurobiology, Tongji Medical College, Huazhong University of Science and Technology, Wuhan, Hubei, P.R. China; 2 Basic Medical Research Centre, Medical College of Nantong University, Nantong, Jiangsu, P.R. China; Indiana University School of Medicine, UNITED STATES

## Abstract

**Aims:**

Previous studies have demonstrated that expression of the TRPM7 channel, which may induce delayed cell death by mediating calcium influx, is precisely regulated. However, functional regulation of TRPM7 channels by endogenous molecules has not been elucidated. The proinflammatory cytokine IL-6 contributes to regulation of Ca^2+^ influx in cerebral ischemia, but the role of IL-6 in regulating TRPM7 functioning is unknown. Thus, we here investigated the interaction between IL-6 and TRPM7 channels and the relevant mechanisms.

**Materials and Methods:**

Using whole-cell patch-clamping, we first investigated the effect of IL-6 on TRPM7-like currents in primary cultured cortical neurons. Next, TRPM7-overexpressing HEK293 cells were used to confirm the effect of IL-6/sIL-6R on TRPM7. Finally, we used specific signaling pathway inhibitors to investigate the signaling pathways involved.

**Results:**

IL-6 or IL-6/sIL-6R dose-dependently inhibited inward TRPM7 currents, in both primary cultured neurons and HEK293 cells overexpressing TRPM7. In intracellular Mg^2+^-free conditions, extracellular Ca^2+^ or the α-kinase domain of TRPM7 did not participate in this regulation. The inhibitory effect of IL-6 on TRPM7 could be blocked by specific inhibitors of the JAK2−STAT3 pathway, but not of the PI3K, ERK1/2, or PLC pathways.

**Conclusions:**

IL-6 inhibits the inward TRPM7 current via the JAK2−STAT3 signaling pathway.

## Introduction

Recent studies have demonstrated that the transient receptor potential melastatin 7 (TRPM7) channel, also known as ChaK1 [1], TRP-PLIK [2], and LTRPC7 [3], is a bifunctional protein with both kinase and ion channel activities [2]. TRPM7 contributes to various physiological processes, including Mg^2+^ homeostasis [4], cellular growth, survival, and proliferation [5]. TRPM7 channels are important mechanisms mediating delayed neuronal death and cognitive dysfunctions after transient ischemia. Identification of TRPM7 channels significantly improves our understanding of the molecular events leading to ischemic brain damage and provides promising novel targets for post-ischemic therapeutics treating ischemic brain damage, despite anti-excitotoxicity therapy (AET) [6, 7].

TRPM7 is a ubiquitously expressed, non-selective cation channel that displays outward rectifying characteristics and a reversal potential close to 0 mV. Outward rectification is most likely caused by blocking of inward currents at negative potentials by divalent cations because perfusion with divalent-free extracellular solutions augments inward currents and linearizes the current−voltage (I/V) relationship [8]. Under physiological conditions, the inward currents mediated by TRPM7 are usually very small, but these are increased in the context of decreased extracellular Ca^2+^ [9] or pH [10]. In addition to inhibition of the channel by intracellular Mg^2+^ and MgATP [3, 11], TRPM7 can be non-selectively inhibited by μM-levels of Gd^3+^, La^3+^, 2-aminoethoxydiphenyl borate (2-APB) [4, 12], and cellular metabolites, such as spermine [13]. We have previously demonstrated that Nerve growth factor (NGF) inhibits TRPM7-like currents in hippocampal neurons through a TrkA pathway [14] and that TRPM7 expression in hippocampal neurons is modulated by ischemia−reperfusion and oxygen–glucose deprivation [14, 15]. Specific agonists or inhibitors of TRPM7 have not been discovered to date, and the gating and regulatory mechanisms of TRPM7 channels have not been fully elucidated.

A recent report has indicated that the proinflammatory cytokine IL-6 contributes to the inflammatory and neurotrophic aspects of cerebral ischemia [16]. In the acute phase of cerebral ischemia, IL-6 functions as an inflammatory cytokine, while in the subacute and late phases, it contributes to neuroprotection [16]. Research has also indicated that IL-6 may modulate Ca^*2+*^ influx by NMDA attack [17] or L-type Ca^2+^ channels [18]. Therefore, IL-6 may be an important endogenous regulator of Ca^2+^ influx and excitotoxicity in ischemic injury.

The physiological role of IL-6 is mediated by the signal-transducing component of the IL-6 receptor (IL-6R), viz., the gp130 subunit. IL-6 can effectively signal via membrane-bound IR-6R or soluble IR-6R (sIL-6R), which both bind to the ubiquitously expressed gp130 subunit [19]. Such binding activates the downstream JAK−STAT pathway, which then regulates the transcription of target genes [16]. IL-6 primarily activates the classical JAK−STAT signaling pathway, but also activates Erk1/2, p38-MAPK, and PI3-kinase/Akt pathways. [20–22]. During IL-6 trans-signaling, the IL-6 α-receptors functions to increase cell sensitivity to the cytokine. However, the soluble α-receptors (sIL-6Rα) lack transmembrane and cytoplasmic components [23]. Moreover, expression of IL-6Rα is restricted and tightly regulated in vivo. sIL-6Rα and soluble gp130 (sgp130) are both present in human serum [16, 24] and sIL-6R is a potent agonist of IL-6 signaling in many cell types [25, 26], partly because the soluble receptor retains the ability to undergo IL-6-dependent gp130 dimerization. sIL-6R function can be suppressed by IL-6R antibodies [19, 27].

Both IL-6 and TRPM7 are up-regulated in cerebral ischemia [6, 16] and are known to contribute to neuronal damage, but it is unknown whether these two molecules interact during this process. Therefore, the present study aimed to investigate the effects of IL-6 on TRPM7 functioning in cerebral ischemia−reperfusion injury, and to elucidate the relevant mechanisms.

## Materials and Methods

### Ethics statement

All animals were handled according to the Council for International Organizations of Medical Sciences on Animal Experimentation and the Huazhong University of Science and Technology guidelines for the use of laboratory animals. The protocol was approved by the Committee on the Ethics of Animal Experiments of Tongji Medical College, Huazhong University of Science and Technology. All surgery was performed according to approved protocols, and all efforts were made to minimize animal distress.

### Cell culture

Primary cortical neuronal cultures were prepared from 18-day pregnant Sprague−Dawley rat embryos obtained from Tongji Medical College, Huazhong University of Science and Technology, according to a previously described procedure [15]. Living embryos were removed from the pregnant rat by caesarian section under pentobarbital sodium anesthesia. Brains were removed from the embryos, and the cerebral cortex was dissected in D-Hanks’ balanced salt solution (pH 7.3), followed by digestion in 0.25% trypsin (Gibco BRL, Grand Island, New York, USA) at 37°C for 15 min. Trypsinization was terminated using DMEM (HyClone, Logan, UT) with 10% fetal bovine serum (FBS; Gibco). Next, the cortex samples were triturated using a fire-polished Pasteur pipette and filtered through a 100-μm mesh. Dissociated cells were plated at a density of 2 × 10^6^ cells/mL onto 0.1 mg/mL poly-L-lysine- coated (Sigma, St. Louis, MO, USA) coverslips and maintained in DMEM supplemented with 10% FBS. After 6 h, the culture medium was completely replaced with maintenance medium containing 97% Neurobasal (Invitrogen, Carlsbad, CA, USA), 2% B27 (Invitrogen), and 1% L-glutamine (Invitrogen), and received half-changes every 3 days. Cultured neurons were utilized for experiments after 10 days.

TRPM7-overexpressing cell lines included HEK293-TRPM7/WT cells (expressing full-length TRPM7) and HEK293-TRPM7/△KIN cells (expressing kinase domain-truncated TRPM7), which were obtained as previously described [15]. Both types of HEK293 cells were grown on glass coverslips in DMEM supplemented with 10% FBS, 0.1% blasticidin, and 0.1% hygromycin.

### TRPM7 current recordings in neurons

Patch-clamp experiments were performed in the whole-cell configuration at 21−25°C using neurons grown on glass coverslips and maintained in an Mg^2+^-free extracellular solution of the following composition (in mM): 140 NaCl, 5.4 KCl, 33 Glucose, 25 HEPES and 1.3 CaCl_2_, with pH adjusted to 7.4 using NaOH. Patch electrodes were fabricated using a PUL-2 micropipette puller (700C, Japan.). The resistance was 3–5 MΩ when filled with Mg^2+^-free internal solution of the following composition (mM): 140 CsCl, 10 HEPES, 2 TEACL, 5 EGTA, and 1 CaCl_2_, with pH adjusted to 7.4 using CsOH. High-resolution current recordings were acquired using a Multiclamp700B patch-clamp amplifier system (Axon Instruments, Foster City, CA, USA). Following whole-cell formation, 2-s voltage ramps between -80 mV and +100 mV were delivered from a holding potential of -60 mV. Currents were filtered at 2 kHz and digitized at 10 kH using a Digidata 1440 data acquisition system (Axon Instruments, USA). Capacitive currents and series resistance were determined and corrected prior to each voltage ramp, using the automatic capacitance compensation of the Multiclamp 700B. Voltage-gated Na^+^ and Ca^2+^ channels were blocked using 1 mM tetrodotoxin (TTX) and 5 mM nimodipine, respectively. For some cells, Ca^2+^-free extracellular solution was used to abolish the inhibitory role of Ca^2+^ on TRPM7 currents. Inward TRPM7 currents are relatively small; therefore, we observed the effects of IL-6 on TRPM7 currents using a normalized current obtained by calculating current values at each voltage divided by the inward current value for the control condition at +100 mV.

### TRPM7 current recordings in HEK293 cells

TRPM7 expression was induced in HEK293 cells by adding 1 μg/mL tetracycline to the culture medium; whole-cell patch-clamp experiments were performed 18−48 h later. Following whole-cell formation, the current-voltage relationships were obtained using a 500-ms voltage ramp pulse, applied from -100 mV to 100 mV; the holding potential was set at 0 mV. The extracellular solution was the same as that used for neurons, except that the concentration of Ca^2+^ was 0 mM. The pipette solution contained (in mM): 145 Cs-methanesulfonate, 5 EGTA, 1 CaCl_2_, and 10 HEPES. All voltages were corrected for a liquid junction potential of 10 mV between external and internal solutions.

### Compounds

Pharmacological compounds used in this study included recombinant human IL-6 (Preprotech, London, UK), recombinant human IL-6R (Preprotech), rabbit monoclonal antibody against IL-6R (R&D Systems, Inc. Minneapolis, MN, USA), and goat polyclonal antibody against TRPM7 (Santa Cruz Biotechnology, Santa Cruz, CA, USA). The following compounds were purchased from Sigma−Aldrich (St. Louis, MO, USA): the JAK2 inhibitor AG490, the PI3K inhibitor wortmannin, the PLC inhibitor U73122, TEACL, TTX, nimodipine, CsCl, choline chloride, spermine, GdCl3, and 2-APB. The STAT3 inhibitor HO-3867 and the Erk inhibitor PD98059 were purchased from Selleck Chemicals (Houston, TX, USA). Dimethyl sulfoxide (DMSO) was used to dissolve hydrophobic inhibitors, including 2-APB, AG490, PD98059, HO-3867, and U73122, with resulting final concentrations of DMSO of less than 0.1% (v/v).

### Statistical analyses

Statistical values of averaged data are given as mean ± S.E.M. Comparisons between two groups were analyzed using Student’s *t*-test, other comparisons were made using one-way ANOVA followed by Dunnett’s test. Statistical significance was set as p < 0.05.

## Results

### Inhibition of inward TRPM7-like currents by IL-6 in cultured primary cortical neurons

**Fig 1:**
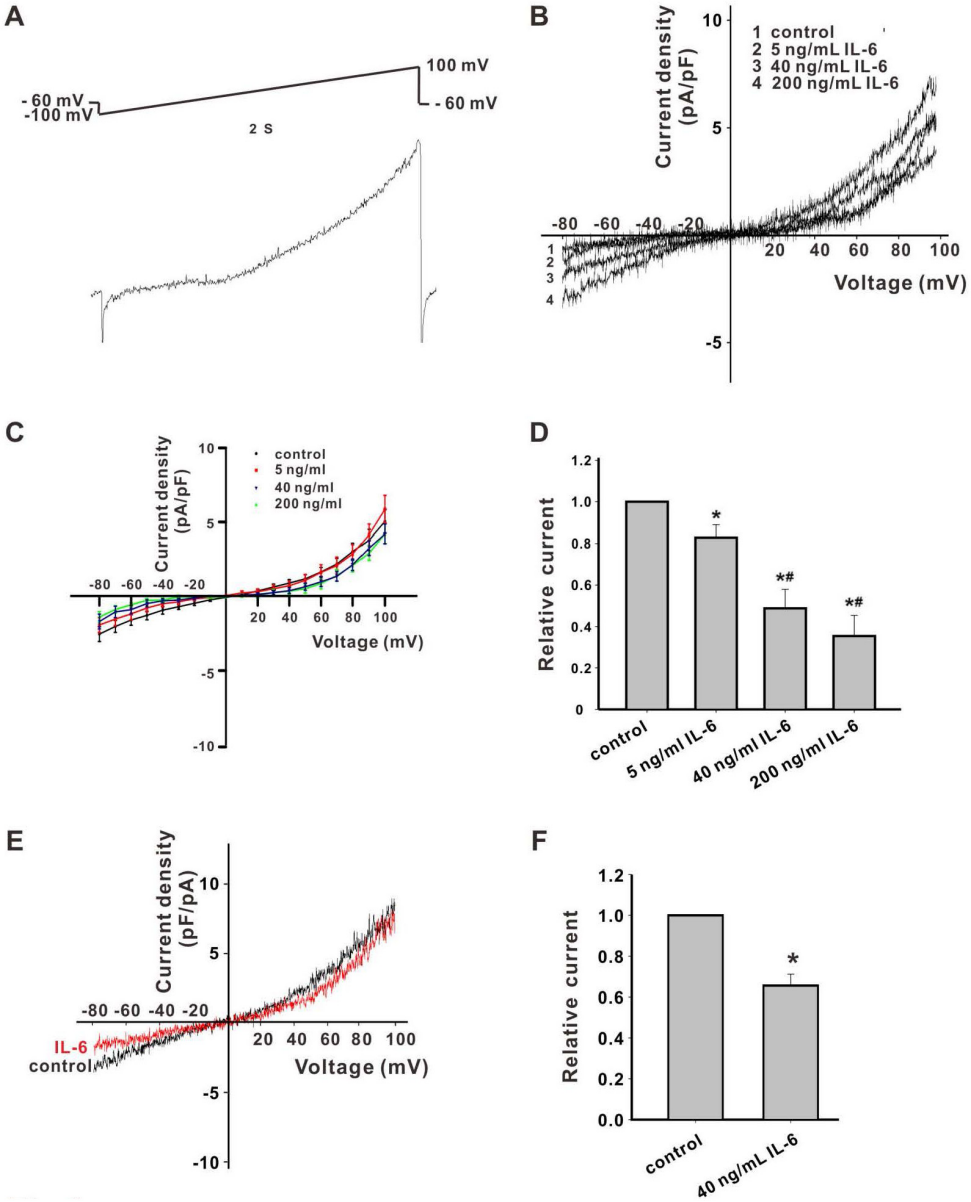
Inhibitory effect of IL-6 on TRPM7-like in cortical neurons. (A) The voltage ramp protocol invoking TRPM7-like currents, and a representative typical current in neurons. (B) The representative TRPM7-like current in cortical neurons perfused with IL-6 at different concentrations in normal extracellular solution. (C) Current−voltage relationships of TRPM7-like currents perfused with IL-6 at different concentrations. *p < 0.05 vs. control. (D) Dose-dependent reduction of the TRPM7-like currents in neurons at -80 mV by IL-6 at concentrations of 8 ng/mL, 40 ng/mL, and 200 ng/mL respectively (n = 9, * p < 0.05 vs. control, # p < 0.01 vs. control). (E) Typical TRPM7-like current in cortical neurons perfused with IL-6 in Ca^2+^-free extracellular solution. (F) Inhibition of inward TRPM7-like currents in cortical neurons perfused with IL-6 in Ca^2+^-free extracellular solution.

TRPM7-like currents can be identified by their biophysical characteristics, even without applying specific agonists during the electrophysiological recordings [3, 13]. TRPM7-like currents were generated in rat cortical neurons using a previously validated protocol [4]. Under whole-cell configuration, typical outward rectified TRPM7-like currents could be recorded in neurons (Fig 1A). In order to clarify the effects of IL-6 on TRPM7-like currents, I/V relationships of TRPM7-like currents were observed in neurons perfused with normal extracellular solution, containing varying concentrations of IL-6 (Fig 1B and 1C). Compared with normal group, the inward TRPM7-like currents were reduced by 17.1 ± 6.0%, 51.2 ± 9.0%, and 64.8 ± 10.1% when perfused with 8 ng/mL, 40 ng/mL, and 200 ng/mL IL-6, respectively (p < 0.05; Fig 1D). IL-6 induced no marked changes in outward currents. Using divalent-free extracellular solution, the inhibition of inward TRPM7-like currents by 40 ng/mL IL-6 was 34.4 ± 5.6% (p < 0.05), which was not significantly different from recordings with normal extracellular solution containing 40 ng/mL IL-6. Outward TRPM7-like currents were not affected by IL-6, and the recording also did not significantly differ from those recorded in normal extracellular solution containing IL-6 (Fig 1E and 1F). These data indicate that IL-6 inhibited inward, but not outward TRPM7-like currents, and that the effects of IL-6 on TRPM7-like current are Ca^2+^-independent.

### IL-6 regulates TRPM7 currents through its α-receptor

Next, we performed experiments using heterologous TRPM7-overexpressing HEK293 cells. Wild-type TRPM7 expression was induced by exposure to tetracycline for 18−48 h, and cells were then used for TRPM7 current recordings. After membrane rupture under the whole-cell configuration, both inward and outward TRPM7 currents were time-dependently increased using pipettes filled with internal Mg^*2+*^-free solution (S1A and S1B Fig). Inward TRPM7 currents in HEK293 cells could be inhibited at -100 mV by 10 μM Gd^3+^ at -100 mV (86.9 ± 9.9%, p < 0.05), but not at +100 mV (S1C and S1D Fig). Moreover, the TRPM7 inward current at -100 mV could be markedly inhibited by 2-APB, by 85.3 ± 4.1%, while the outward current at +100mV was inhibited by only 9.6 ± 4.5% (S1E and S1F Fig). These results are consistent with those of a previous report on the characteristics of TRPM7 channel activity in neurons and other cells [14, 28].

**Fig 2:**
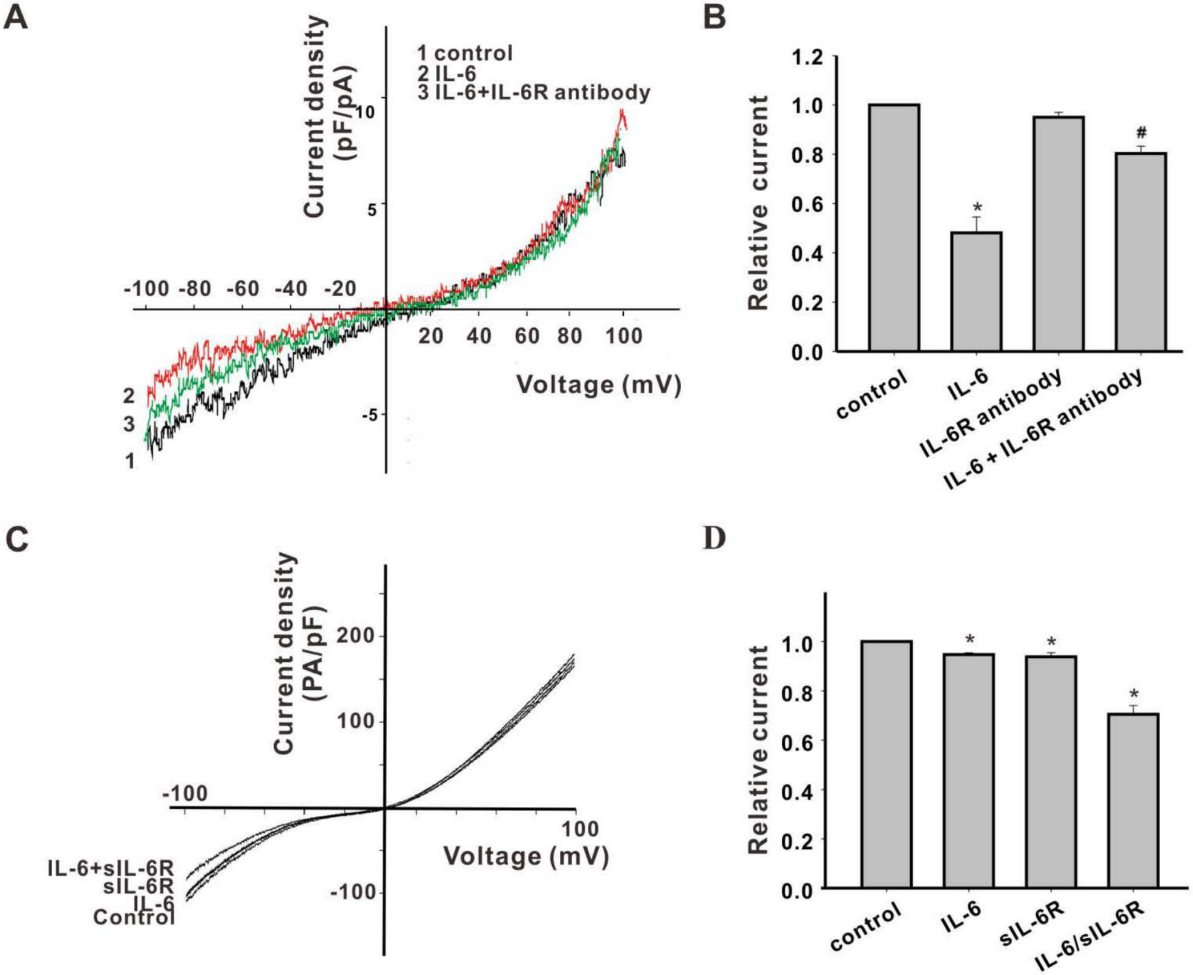
IL-6R-mediated IL-6 inhibition of TRPM7 inward currents. (A) Representative raw traces of TRPM7 current in cortical neurons perfused with IL-6R antibodies in Ca^2+^-free extracellular solution. (B) The inhibition of the TRPM7-like inward current by IL-6 was weakened in the presence of IL-6 receptor antibodies. (C) Representative raw traces of TRPM7 current in HEK293-TRPM7/WT cells perfused with IL-6 or sIL-6R only, or IL-6/sIL-6R, in Ca^2+^-free extracellular solution. (D) Co-treatment with IL-6 and sIL-6R produced maximal inhibition of inward TRPM7 currents in HEK293-TRPM7 cells.

In order to investigate whether IL-6 regulates TRPM7 via an interaction with IL-6R or via direct binding, we conducted whole-cell patch-clamp experiments using IL-6R blocking antibody in neurons and using sIL-6R in HEK293-TRPM7 cells, respectively. Inhibition of IL-6 on inward TRPM7-like currents in neurons could be blocked by the IL-6R antibody (p *<* 0.05; Fig 2A and 2B). Furthermore, in HEK293 cells, TRPM7 currents were inhibited more markedly by IL-6 combined with sIL-6R (19.1 ± 3.0%) than by IL-6 alone (5.4 ± 0.7%) or by sIL-6R alone (6.1 ± 1.9%; p < 0.05; Fig 2C and 2D). These results confirmed that IL-6 inhibits inward TRPM7 currents through its α-receptor.

### IL-6/sIL-6R could inhibit the TRPM7 inward, but not the outward current in TRPM7-overexpressing HEK293 cells

**Fig 3:**
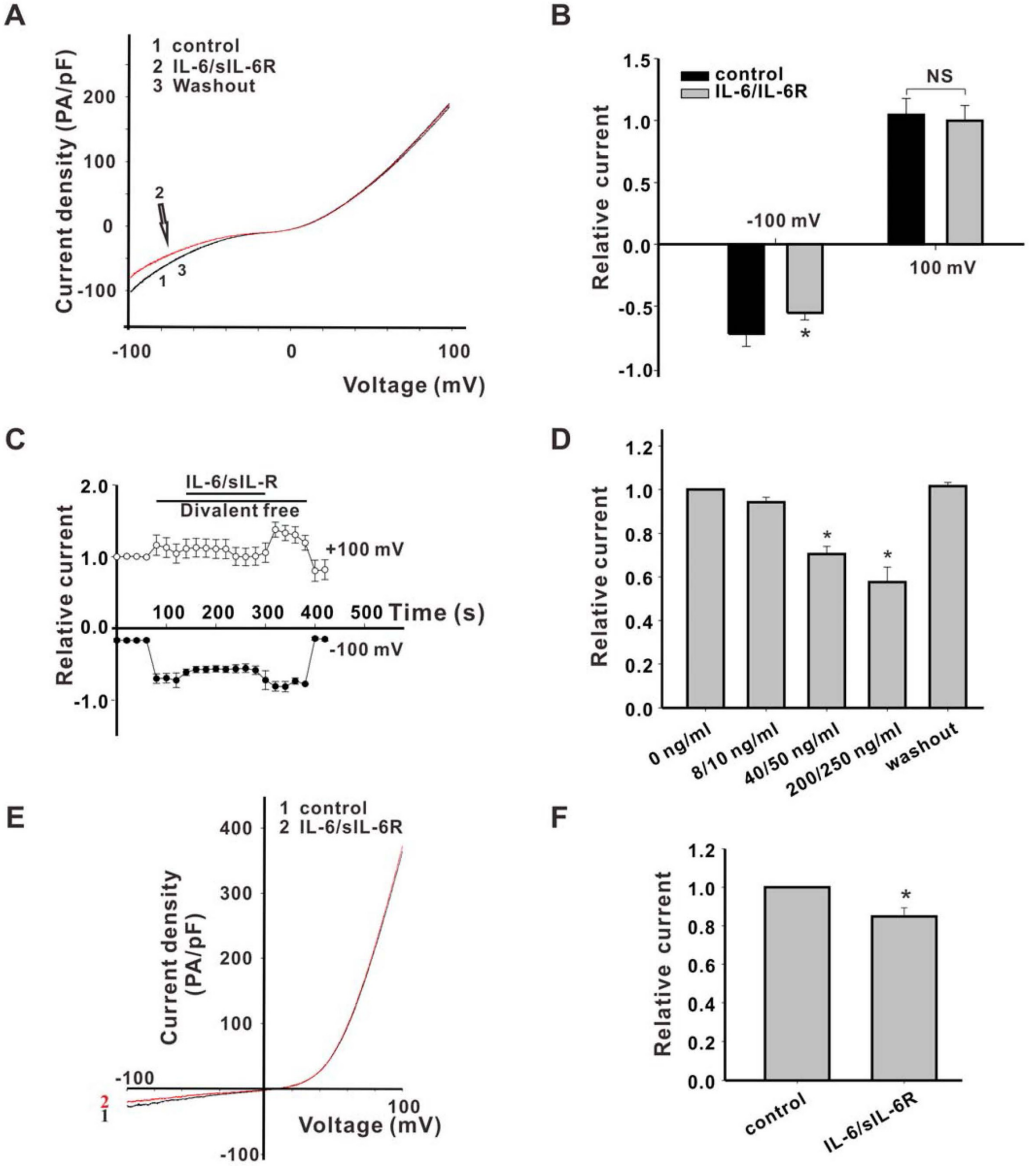
Inhibition of inward TRPM7 current by IL-6/sIL-6R in WT HEK293-TRPM7 cells. (A) Typical TRPM7 currents in WT HEK293-TRPM7 cells treated with 20 ng/mL IL-6 combined with 25 ng/mL sIL-6R in divalent ion-free extracellular solution. (B) Inhibition of TRPM7 inward, but not outward currents, in WT HEK293-TRPM7 cells by IL-6/sIL-6R. All current values were normalized to an inward current value of controls at +100 mV. (n = 6, * p < 0.05). (C) Rapid and reversible inhibition of TRPM7 currents by IL-6 combined with sIL-6R. (D) Dose-dependent reduction of the TRPM7 currents at -80 mV by 8 ng/mL IL-6 combined with 10 ng/mL sIL-6R, 40 ng/mL IL-6 combined with 50 ng/mL sIL-6R, or 200 ng/mL IL-6 combined with 250 ng/mL sIL-6R, respectively. (n = 9, * p < 0.05 vs. control, # p < 0.01 vs. control). (E) A typical TRPM7 current in TRPM7-HEK293 cells perfused with normal extracellular solution. (F) Inhibition of TRPM7 currents by IL-6/sIL-6R in normal extracellular solution in TRPM7-HEK293 cells.

Since IL-6R expression is low in HEK293 cells and IL-6 acts as an agonist of sIL-6R, sIL-6R was used to amplify the effect of IL-6. After pre-incubation with sIL-6R for 20 s, the inward, but not outward, TRPM7 currents were inhibited by IL-6 in combination with sIL-6R (IL-6/sIL-6R), which was consistent with our findings in neurons (Fig 3A and 3B). Fig 3C shows that the inhibition of inward TRPM7 currents by IL-6 was rapid and reversible. The effect of IL-6/sIL-6R on TRPM7 was not altered by removing Ca^2+^ from the extracellular solution, indicating that the effect is not Ca^2+^-dependent (Fig 3E and 3F). Furthermore, we found that IL-6/sIL-6R did not block the inward currents at concentrations of 8 ng/ml IL-6 combined with 10 ng/ml sIL-6R (at -100 mV), but this effect was evident at higher concentrations (40 ng/ml IL-6 combined with 50 ng/ml sIL-6R; 25.4 ± 5.9%, n = 6, p < 0.05; and 200 ng/ml IL-6 combined with 250 ng/ml sIL-6R; 42.4 ± 6.9%, n = 4, p < 0.05; Fig 3D).

### The α-kinase domain of TRPM7 is not involved in the regulation by IL-6

**Fig 4:**
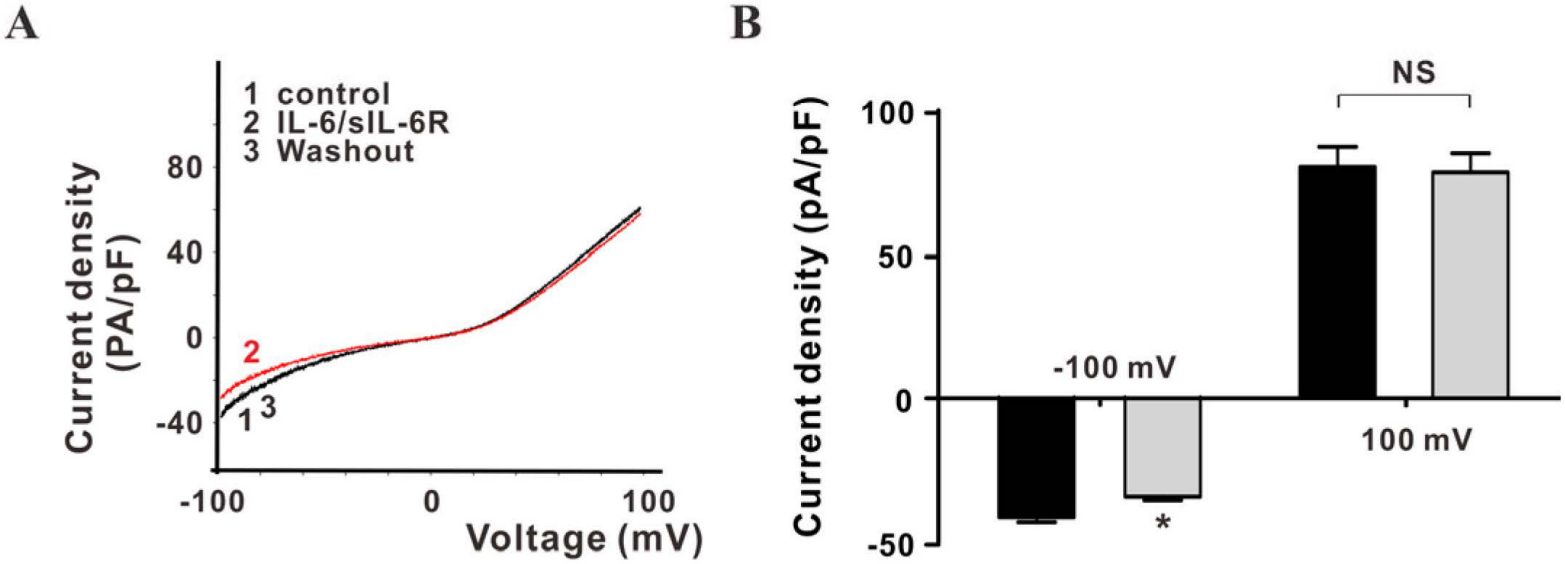
TRPM7 α-kinase does not contribute to the inhibitory role of IL-6 on the inward TRPM7 current. (A) Representative traces of TRPM7 currents in HEK293 cells expressing Δkin-TRPM7 channels prior to (control) and immediately after application of IL-6/sIL-6R. (B) Inhibition of TRPM7 inward, but not outward currents, in HEK293-Δkin cells by IL-6/sIL-6R. All current values were normalized to the control inward current at -100 mV. (n = 12, * p < 0.05).

In order to investigate the role of the α-kinase domain of TRPM7 in regulation by IL-6, we performed experiments using HEK293 cells that overexpress an α kinase domain-truncated TRPM7. The current in HEK293 cells overexpressing this Δkinase TRPM7 (HEK293-Δkin) generally had a markedly smaller amplitude and was more linearized than that observed in wild-type HEK293-TRPM7 cells under the same conditions (Fig 4A). In HEK293-Δkin cells, inhibition of TRPM7 inward currents at -100 mV by 40 ng/ml IL-6 combined with 50 ng/ml sIL-6R was 19.4 ± 3.2%, while the outward TRPM7 currents could be not inhibited at +100 mV by IL-6/sIL-6R. The effect of IL-6/sIL-6R on HEK293-Δkin cells was comparable to the effects observed in wild-type cells. Therefore, IL-6 regulation of TRPM7 channel function does not appear to be exerted via altered α kinase activity (Fig 4B).

### IL-6 inhibits TRPM7 currents through a JAK2−STAT3 signaling pathway

**Fig 5:**
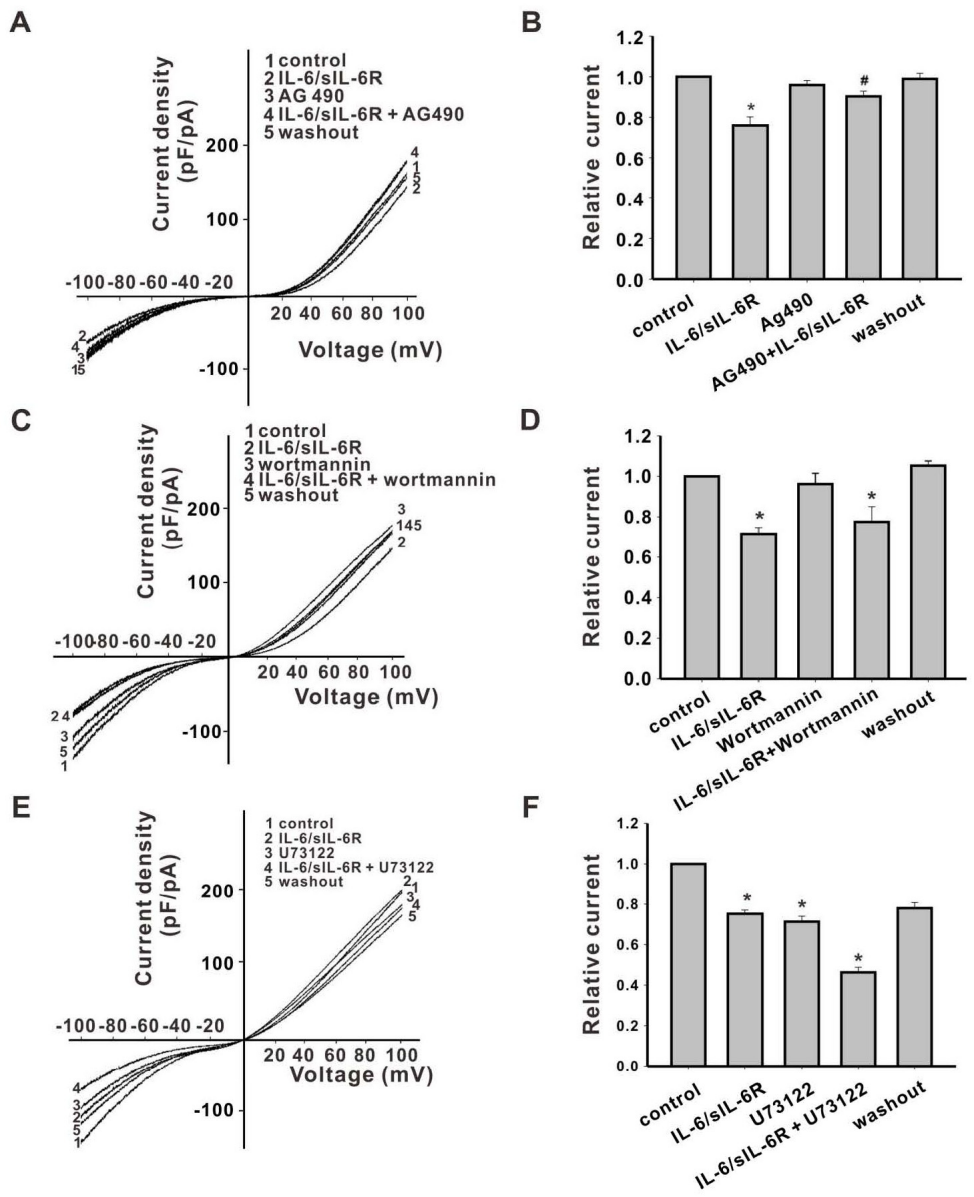
The inhibitory effect of IL-6/IL-6R on TRPM7 currents is blocked by the JAK2 inhibitor AG490, but not by inhibitors of PI3K and PLC. (A) Currents recorded when cells were perfused with divalent ion-free extracellular solution, IL-6/sIL-6R, AG490, or AG490 combined with IL-6/sIL-6R, respectively. (B) The inhibitory role of IL-6 on TRPM7 inward currents could be blocked by the JAK2 inhibitor AG490 (n = 11) (C) Current traces recorded with perfusion of divalent ion-free extracellular solution, IL-6/sIL-6R, wortmannin, or wortmannin combined with IL-6/sIL-6R, respectively. (D) Relative currents normalized to the TRPM7 current at -100 mV and recorded during perfusion of divalent ion-free extracellular solution. The specific PI3K inhibitor wortmannin did not block the effect of IL-6 on TRPM7 inward currents at -100 mV (n = 6). (E) Current traces recorded during perfusion of divalent ion-free extracellular solution, IL-6/sIL-6R, U73122, or U73122 combined with IL-6/sIL-6R, respectively. (F) Relative currents normalized to TRPM7 currents recorded with perfusion of divalent ion-free extracellular solution. The PLC inhibitor U73122 inhibited TRPM7 inward currents at -100 mV, but did not block the effects of IL-6/sIL-6R (n = 7, * p < 0.05 vs. control, # p < 0.05 vs. IL-6/sIL-6R). All currents were normalized to controls (perfusion with divalent ion-free extracellular solution) at -100 mV.

To investigate the possible signaling pathways involved in IL-6 regulation of TRPM7 activity, several specific inhibitors of IL-6 signaling were applied individually, 30 s prior to electrophysiological recording, and were maintained during recordings in TRPM7-overexpressing HEK293 cells. Their effects on currents were compared against identical conditions lacking the inhibitor. The JAK2 inhibitor AG490 had no effect on TRPM7 currents, but it reduced the inhibitory effect of IL-6 (40 ng/mL) combined with sIL-6R (50 ng/mL; 24.0 ± 4.2%) on normalized inward TRPM7 currents at -100 mV. The effect of AG490 was reversible, as inhibition by IL-6/sIL-6R occurred after washout (Fig 5A and 5B). The inhibition of PIP2 generation by a high concentration of wortmannin (10 μM), a specific inhibitor of PI3K, decreased the TRPM7 current. However, a low concentration of wortmannin (100 nM), which did not alter TRPM7 currents when applied alone (S2 Fig), had no effect on IL-6/sIL-6R regulation of the inward TRPM7 current (Fig 5C and 5D). Inward TRPM7 currents could be reduced by U73122, an inhibitor of PLC (inhibited by 28.7 ± 2.7% at -100 mV), and inward currents were further attenuated when applied in combination with IL-6/sIL-6R (inhibited by 53.7 ± 2.5% at -100 mV). Therefore, TRPM7 channel activity could be differently regulated through JAK2 and PLC pathways (Fig 5E and 5F).

**Fig 6:**
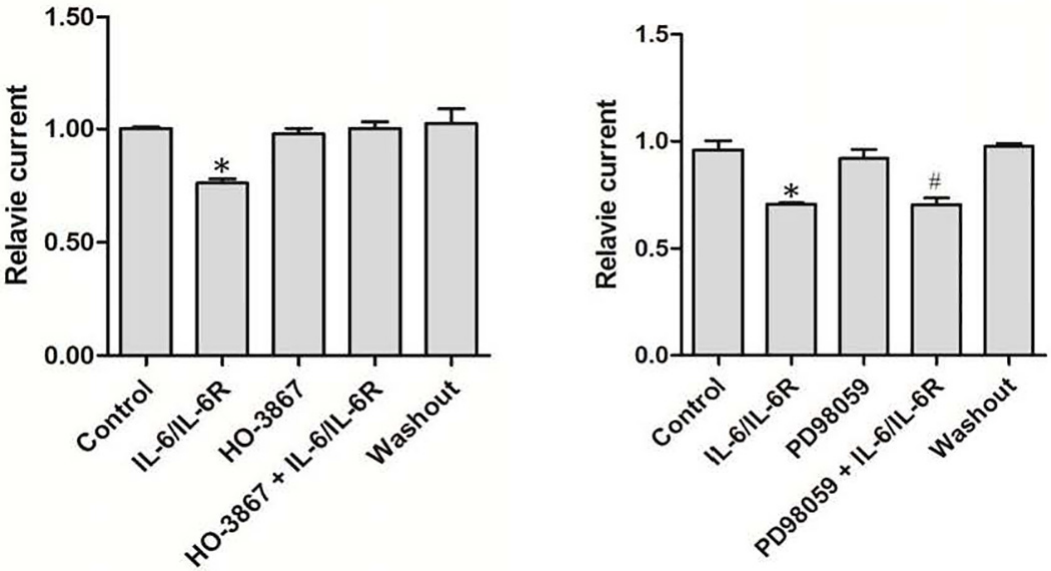
The inhibitory effect of IL-6/IL-6R on TRPM7 currents is blocked by the STAT3 inhibitor HO-3867, but not by the inhibitor MAPK signaling pathway. (A) Relative currents normalized to TRPM7 currents recorded during perfusion with divalent-free extracellular solution. The specific STAT3 inhibitor HO-3867 did not inhibit TRPM7 inward currents at -100 mV, but blocked the effect of IL-6/sIL-6R (n = 3, * p < 0.05 vs. control, # p < 0.05 vs. IL-6/sIL-6R). (B) The specific MAPK−MEK inhibitor PD98059, did not inhibit TRPM7 inward currents at -100 mV, and did not block the effect of IL-6/sIL-6R (n = 3, * p < 0.05 vs. control). All currents were normalized to controls at -100 mV.

To further explore the downstream signaling pathways, specific inhibitors of JAK2−STAT and MAPK signaling pathways were also applied with the Ca^2+^-free extracellular solution containing IL-6/sIL-6R. The STAT3 inhibitor HO-3687 had no effect on TRPM7 inward currents, but it reduced the inhibitory effect of IL-6 at 40 ng/mL combined with sIL-6R at 50 ng/mL on normalized inward TRPM7 currents at -100 mV (Fig 6A). On the other hand, the MAPK inhibitor PD98059, which had no effect on the TRPM7 inward current, also did not reverse the effect of IL-6/IL-6R regulation of TRPM7 (Fig 6B).

## Discussion

In the present study, we demonstrated that IL-6 dose-dependently inhibited TRPM7 inward currents in cultured primary cortical neurons as well as in HEK293 cells overexpressing TRPM7. The observed inhibitory effect of IL-6 on TRPM7 was independent of extracellular [Ca^2+^], intracellular [Mg^2+^], and TRPM7 α-kinase activity. Additionally, TRPM7 was regulated by both the membrane-bound and soluble IL-6R through the downstream activation of JAK2.

IL-6-type cytokines play an important role in a variety of pathophysiological CNS conditions [29]. It is known that IL-6-type cytokines can produce receptor-mediated intracellular signaling that interferes with ion transport. For example, leukemia inhibitor factor activates rabbit cardiac L-type Ca^2+^ channels [30], while IL-6 inhibits L-type Ca^2+^ channels in cerebellar granule neurons [18] and prevents metabotropic glutamate receptor-activated Ca^2+^ signaling in cerebellar Purkinje neurons [31]. Li *et al*. reported that IL-6 inhibits voltage-gated Na^+^ channel activity in rat spinal cord neurons [32]. Xia *et al*. reported that IL-6 regulates voltage-gated Na^+^ channels in cortical neurons, thereby mediating a neuroprotective effect [33]. We report the novel finding that IL-6 also inhibits the activity of the TRPM7 channel, which is also a Ca^2+^ permeable channel, contributing to anoxic neuronal death [6]. In ischemia−reperfusion injury, IL-6 produced during the acute phase acts as a neurotrophic mediator between the subacute and prolonged phases of injury [16]. Additionally, TRPM7 channels, but not NMDA receptors, contribute to the late phase of ischemia−reperfusion injury [6]. Therefore, we hypothesized that the inhibition of TRPM7 by IL-6 promotes neuronal survival in ischemia−reperfusion injury.

Signaling mediated by the membrane-bound IL-6R and sIL-6R is referred to as classical cis-signaling and trans-signaling, respectively [14]. IL-6Rs have a common structural basis and interact with the signal transducing co-receptor gp130 to couple to downstream JAK−STAT signaling pathways. In this study, we used a neutralizing IL-6R antibody as a pharmacological tool to block the activation of the membrane-bound IL-6R in neurons and sIL-6R in HEK293 cells. The neutralizing IL-6R antibody attenuated the inhibitory effect of IL-6 on TRPM7 currents in primary cultured cortical neurons. Alternatively, we used exogenous sIL-6R to induce IL-6 trans-signaling [4] in the HEK293 cell line, which rarely expresses the membrane-bound IL-6R (data not shown). Consistent with our observation in cortical neurons, the neutralizing IL-6R antibody blocked the ability of exogenous IL-6 and sIL-6R to inhibit TRPM7 currents in HEK293 cells. Together, these results suggested that IL-6 inhibits the TRPM7 current through its receptor-mediated cis-signaling or trans-signaling mechanisms.

The binding of IL-6 to its specific receptor causes the recruitment of two gp130 co-receptors to form the IL-6-IL-6R-gp130 complex [34]. This subsequently leads to the activation of tyrosine kinases JAK1, JAK2, and TYK2, and the phosphorylation of STAT1 and STAT3 [29]. In this study, we show that AG490, a specific JAK2 inhibitor, and HO-3867, a specific STAT3 inhibitor, abrogates the inhibitory effect of IL-6 on TRPM7 current. This finding is consistent with several reports that demonstrate the role of JAK2 in the regulation of ion-channel activity [35–38]. Because IL-6 modulates TRPM7 current with fast kinetics, we speculate that the effect of IL-6 signaling on TRPM7 may result from JAK2−STAT3 mediated phosphorylation of TRPM7, or another direct interaction between IL-6R signal transducers and TRPM7.

The canonical gp130/JAK2/STAT3 IL-6 signaling pathway regulates several complex cellular processes, including gene activation and cellular proliferation and differentiation [39–40]. STAT3-dependent gene expression leads to the upregulation of the suppressor of cytokine signaling 3, (SOCS3) an important negative regulator of IL-6 signaling [41]. Jin *et al*. reported that deletion of TRPM7 results in the loss of STAT3 activity in thymic medullary cells, which disrupts embryonic development and thymopoiesis [42]. In another study, Yee *et al*. demonstrated that *SOCS3A* mRNA levels are elevated in TRPM7 loss-of-function Zebrafish sweetbread (swd) mutants, and that the Mg^2+^-sensitive SOCS3a pathway links TRPM7 function to growth factor-and cytokine-induced signaling [43]. These studies implicate TRPM7 as an important regulator of IL-6 signaling. Conversely, we showed here that IL-6 signaling can also provide feedback to TRPM7 channel activity, suggesting that crosstalk occurs between IL-6 signaling and TRPM7. Further studies are needed to determine whether SOCS3 participates in the regulation of TRPM7 channel expression and function.

IL-6 can also activate PI3K/AKT and MEK/ERK1/2 signaling, as well as other intracellular cascades [20–22]. Wortmannin is widely used as a PI3K inhibitor at a low concentration (10−100 nM), even though it also serves as a PI4K inhibitor at a higher concentration (1−10 μM). In our study, 100 nM wortmannin was selected to prevent PI3K activation by IL-6. This would not affect PI4K, although hydrolysis of its target, PIP2, inactivated the TRPM7 channel. In our previous study, wortmannin at 100 nM did not block the effect of IL-6 on TRPM7. In addition, PD98059, an inhibitor of MEK, could not block the effect of IL-6 on TRPM7 currents. Previously, Runnels *et al*. demonstrated that PLC-coupled receptor agonists, such as choline, lead to the hydrolysis of PIP2 and the inactivation of the TRPM7 channel [44]. It was argued that experiments confirming this phenomenon were confounded by the TRPM7-overexpression system or by coupled PLC receptor agonists. The overexpression of TRPM7 led to the complete inhibition of signaling by PLC and prevented the degradation of PIP2, without affecting muscarinic regulation of TRPM7 [45]. In our study, however, U73122 inhibited TRPM7 inward currents. Furthermore, inhibition achieved by the combination of U73122 and IL-6 was greater than that achieved with U73122 or IL-6 alone. These data suggest that IL-6 may regulate TRPM7 by different pathways than PLC.

In our study, TRPM7 currents were generated using a protocol previously used in neurons and TRPM7-HEK293 cells [4, 14]. Depletion of intracellular Mg^2+^ or extracellular Ca^2+^ did not alter the inhibitory effect of IL-6 on the TRPM7 current. TRPM7 is a known bifunctional protein, with both kinase and ion channel activities. Takezawa *et al*. showed that TRPM7 activity is regulated through its endogenous kinase in a cAMP- and PKA-dependent manner [45], while Matsushita *et al*. demonstrated that neither the TRPM7 current nor its regulation by internal Mg^2+^ was affected by kinase activity or autophosphorylation [46]. In our study, the current recorded from HEK293 cells expressing an α-kinase domain-truncated form of TRPM7 (HEK293-Δkin) was also inhibited by the application of IL-6, although the baseline TRPM7 currents were smaller in HEK293-Δkin cells than in the corresponding wild-type cells. These data indicated that the α-kinase domain of TRPM7 is unlikely to be involved in IL-6-mediated regulation of the TRPM7 channel.

In conclusion, our results suggest that IL-6 rapidly regulates the activity of TRPM7 channels through the JAK2-STAT3 pathway.

## Supporting Information

S1 Fig Characteristics of TRPM7 currents in HEK293 cells overexpressing TRPM7. (A) Time-dependent increase in TRPM7 currents after break-in when perfused with Mg^2+^-free internal solution in whole-cell patch clamp. (B) Both TRPM7 inward and outward currents normalized to control cells at +100 mV could increase during whole-cell patch clamp recording (n = 11). (C) Typical TRPM7 currents when perfused with Gd^3+^ or not. (D) Showing Gd^3+^ could reversibly inhibit both TRPM7 inward currents at -80 mV and outward currents at +100 mV. (E) Typical TRPM7 currents recorded when perfused with divalent-free ECF, 2-APB and washout respectively. (F) Showing both TRPM7 inward currents at -80 mV and outward currents at +100 mV could be inhibited reversibly by 2-APB. (n = 6).
(TIF)

S2 Fig The effect of wortmannin on the TRPM7 current in heterologous TRPM7-overexpressing HEK293 cells. (A) A high concentration of wortmannin (10 μM) inhibited the TRPM7 current (n = 3, * p < 0.05). (B) A relatively low concentration of wortmannin (100 nM) did not inhibit the TRPM7 current. Relative currents normalized to TRPM7 currents recorded during perfusion with divalent ion-free extracellular solution.
(TIF)
