# Supplementary figures and images for: Regulation of TRPM7 Function by IL-6 through the JAK2-STAT3 Signaling Pathway
Source: PLoS One. 2016 Mar 24;11(3):e0152120. doi: 10.1371/journal.pone.0152120 (PMC4806911; doi:10.1371/journal.pone.0152120)

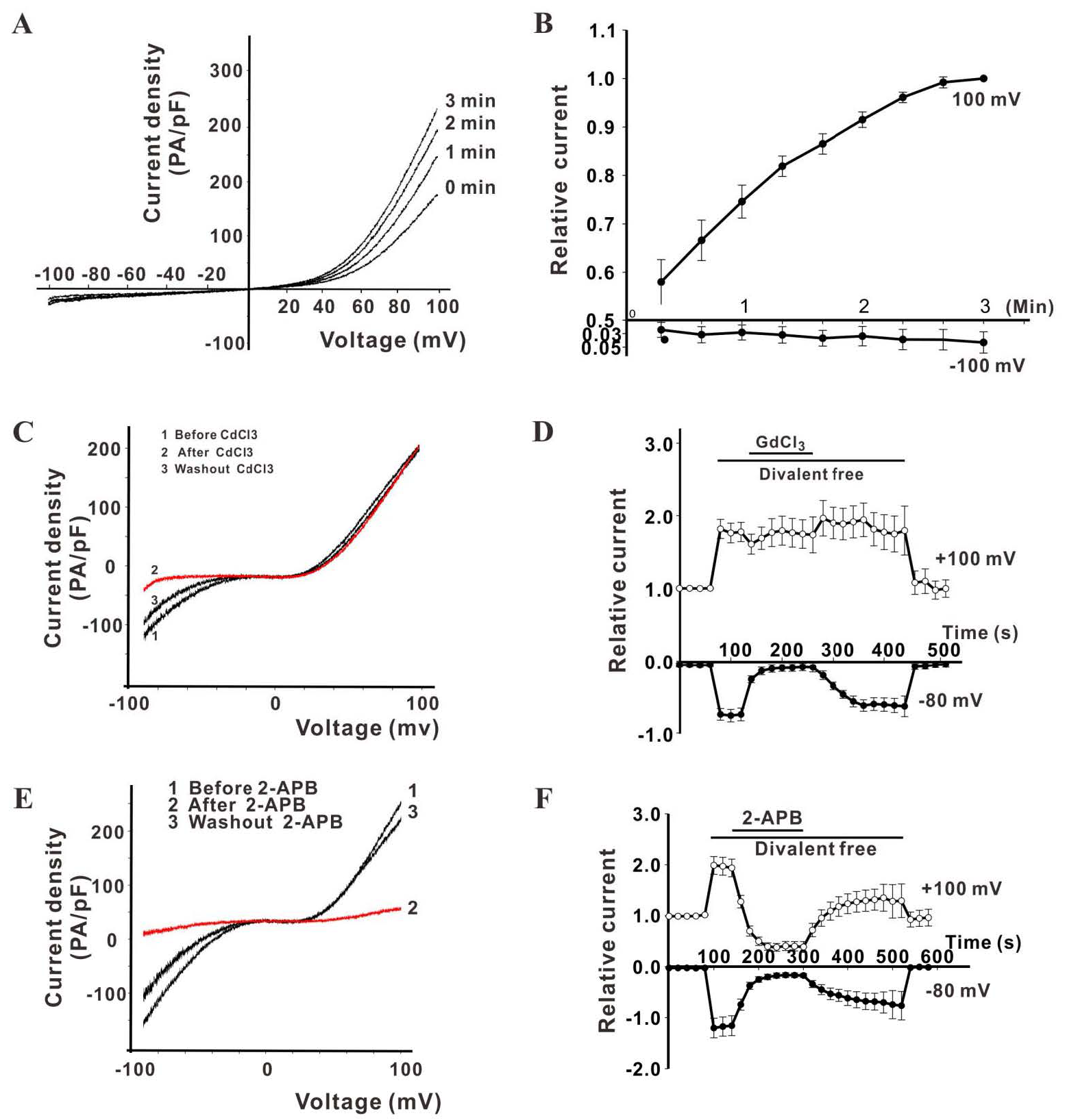

Supplement: S1 Fig — (A) Time-dependent increase in TRPM7 currents after break-in when perfused with Mg2+-free internal solution in whole-cell patch clamp. (B) Both TRPM7 inward and outward currents normalized to control cells at +100 mV could increase during whole-cell patch clamp recording (n = 11). (C) Typical TRPM7 currents when perfused with Gd3+ or not. (D) Showing Gd3+ could reversibly inhibit both TRPM7 inward currents at -80 mV and outward currents at +100 mV. (E) Typical TRPM7 currents recorded when perfused with divalent-free ECF, 2-APB and washout respectively. (F) Showing both TRPM7 inward currents at -80 mV and outward currents at +100 mV could be inhibited reversibly by 2-APB. (n = 6). (TIF) [file pone.0152120.s001.tif]

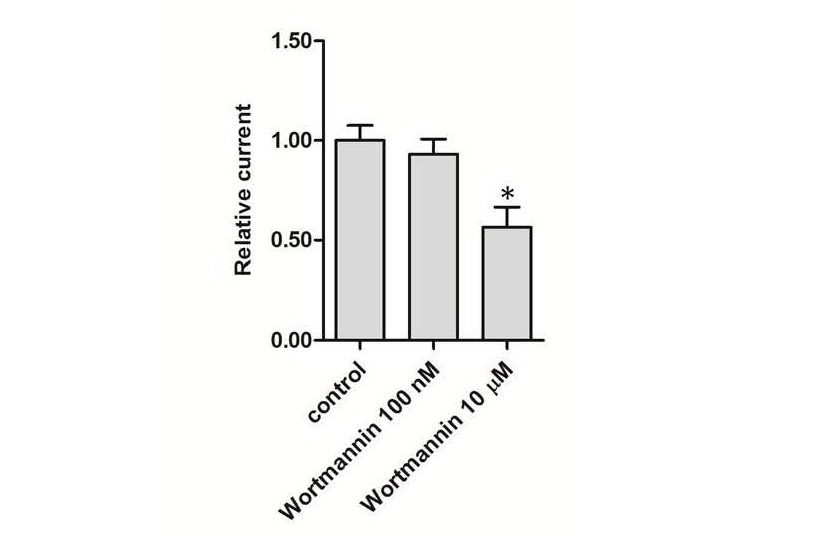

Supplement: S2 Fig — (A) A high concentration of wortmannin (10 μM) inhibited the TRPM7 current (n = 3, * p < 0.05). (B) A relatively low concentration of wortmannin (100 nM) did not inhibit the TRPM7 current. Relative currents normalized to TRPM7 currents recorded during perfusion with divalent ion-free extracellular solution. (TIF) [file pone.0152120.s002.tif]
